# Inverted Pendulum Standing Apparatus for Investigating Closed-Loop Control of Ankle Joint Muscle Contractions during Functional Electrical Stimulation

**DOI:** 10.1155/2014/192097

**Published:** 2014-10-28

**Authors:** John F. Tan, Kei Masani, Albert H. Vette, José Zariffa, Mark Robinson, Cheryl Lynch, Milos R. Popovic

**Affiliations:** ^1^Institute of Biomaterials and Biomedical Engineering, University of Toronto, 164 College Street, Toronto, ON, Canada M5S 3G9; ^2^Toronto Rehabilitation Institute, University Health Network, 520 Sutherland Drive, Toronto, ON, Canada M4G 3V9; ^3^Department of Mechanical Engineering, University of Alberta, 4-9 Mechanical Engineering Building, Edmonton, AB, Canada T6G 2G8; ^4^Glenrose Rehabilitation Hospital, Alberta Health Services, 10230-111 Avenue, Edmonton, AB, Canada T5G 0B7; ^5^Department of Kinesiology, University of Waterloo, 200 University Avenue West, Waterloo, ON, Canada N2L 3G1

## Abstract

The restoration of arm-free standing in individuals with paraplegia can be facilitated via functional electrical stimulation (FES). In developing adequate control strategies for FES systems, it remains challenging to test the performance of a particular control scheme on human subjects. In this study, we propose a testing platform for developing effective control strategies for a closed-loop FES system for standing. The Inverted Pendulum Standing Apparatus (IPSA) is a mechanical inverted pendulum, whose angular position is determined by the subject's ankle joint angle as controlled by the FES system while having the subject's body fixed in a standing frame. This approach provides a setup that is safe, prevents falling, and enables a research and design team to rigorously test various closed-loop controlled FES systems applied to the ankle joints. To demonstrate the feasibility of using the IPSA, we conducted a case series that employed the device for studying FES closed-loop controllers for regulating ankle joint kinematics during standing. The utilized FES system stimulated, in able-bodied volunteers, the plantarflexors as they prevent toppling during standing. Four different conditions were compared, and we were able to show unique performance of each condition using the IPSA. We concluded that the IPSA is a useful tool for developing and testing closed-loop controlled FES systems for regulating ankle joint position during standing.

## 1. Introduction

A number of functional electrical stimulation (FES) systems have been proposed to date that are intended to allow individuals with neurological disorders to stand [1–14], with some of them applying closed-loop control strategies in order to facilitate arm-free standing [1–5, 10, 11, 13, 14]. While some of these systems have been partially successful in facilitating arm-free standing, they may not necessarily become practical applications in the near future due to a number of remaining challenges and limitations. One important challenge is that an optimal control strategy for a closed-loop controlled FES system has not been identified to date [15]. Among other reasons, this can be attributed to the fact that it is very difficult to directly test the isolated performance of an FES control strategy in human subjects. For example, contributions of other body parts that are not controlled by the FES system are oftentimes not foreseeable while differences or changes in a stimulated muscle's capacity can modify or even compromise the performance [15].

The current study is set out to develop a platform for testing different control strategies for a closed-loop FES system for ankle joint control during quiet standing, by isolating this joint's action in the standing posture. For this purpose, we developed a device called Inverted Pendulum Standing Apparatus (IPSA) that consists of a human-size inverted pendulum with a point mass at the height of body center of mass and a standing frame that supports the standing posture. IPSA was developed based on the notion that the dynamics of human stance can be modeled as an inverted pendulum with a point mass at the height of the body's center of mass rotating around the ankle joint [16]. The ankle joint is the primary joint that controls the equilibrium of the body's center of mass regardless of other joints' movement as the ankle joint is the first joint contacting the external environment via rigid feet segments. Thus, controlling the ankle joint is the first priority when controlling the entire body. We therefore developed a platform that focuses on stabilizing the ankle joint. Subjects with and without disability in lower-limb muscles can stand on the IPSA with support of a standing frame. In this condition, the plantar- and dorsiflexors are completely relaxed [17] such that we can test the isolated performance of a given FES control system. By regulating the FES intensity, muscle contractions of the user's plantar- and dorsiflexors are modified, modulating in turn the inclination angle of the inverted pendulum. By means of this setup, only the subject's muscles around the ankle joint are utilized, implying that no other body segments contribute to the behavior of the inverted pendulum. Thus, by assessing the “performance” of the inverted pendulum, we are also able to directly evaluate the performance of the tested FES control strategy.

It should be emphasized that a similar device has been developed by Loram's team [18, 19] with the goal of studying human balance control in healthy people. In addition, we have reported on the use of IPSA in a pilot study, which demonstrated that a PID control strategy can successfully stabilize the human size pendulum via ankle joint control [20].　In light of this, the objectives of the present study were to (1) report on the design of the IPSA and (2) demonstrate the use of the IPSA including principles of experimental setup and execution, including the feasibility of using the IPSA to test various FES controllers. For the latter objective, proportional-derivative (PD) control strategies were used with the goal of regulating the ankle joint position during standing via FES. The PD controller was chosen based on our previous studies suggesting that it can be a good model of the physiological controller for quiet standing [21–23] and that a PD-controlled FES system can ensure stable stance by modulating calf muscle contractions [13].

## 2. Methods

### 2.1. Design of IPSA

**Figure 1:**
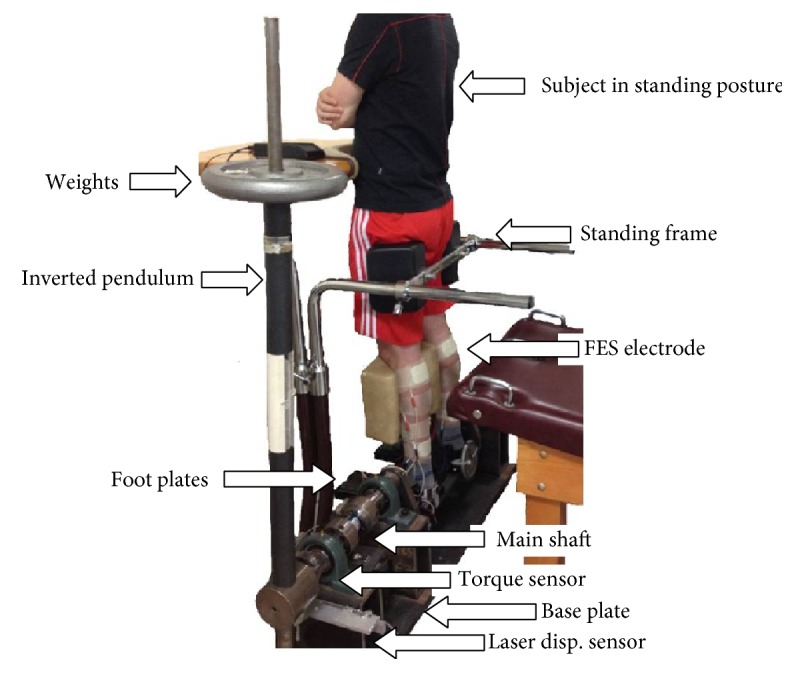
Overall view of the IPSA setup. Depicted are (1) the inverted pendulum (with a shaft fitted to accept additional weights); (2) the standing frame; (3) the subject in a standing posture; (4) foot pedals on which the subject stands; and (5) the main shaft of the IPSA which translates the torque generated by the subject to the pendulum's axis of rotation.

**Figure 2:**
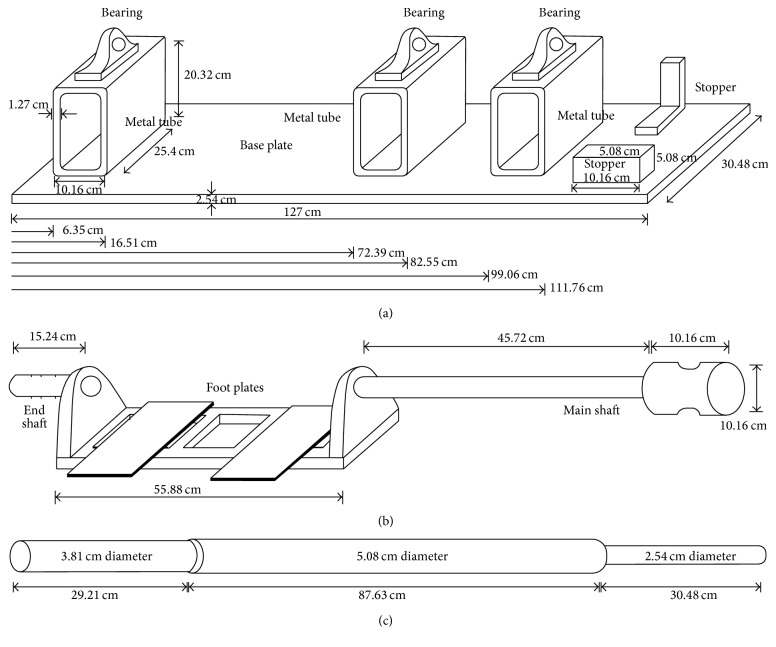
(a) Static components of the IPSA: this figure illustrates the components of the IPSA that remain fixed in place throughout the experiments: the base plate, metal tubes, bearings, and stoppers; (b) moving components of the IPSA: this figure illustrates the main shaft, end shaft, and the foot plates; (c) inverted pendulum: this figure illustrates the dimensions of the inverted pendulum, which locks into place through the two notches on the 4 in. (10.16 cm) end of the main shaft.

Figure 1 shows a photograph of the IPSA setup. The IPSA consisted of (1) an inverted pendulum; (2) a standing frame that is used to secure the subject during the experiments; (3) two connected foot plates on which the subject stands and places his or her feet. Note that the ankle joints are aligned with the center of rotation of the foot plates, which are attached to the inverted pendulum and controlling its movement; (4) a torque transducer that measures the resultant torque at the rotational axis; and (5) a sensor system for measuring the pendulum angle.  Figure 2 shows schematic diagrams of the major components of the IPSA. The static components of the IPSA included a base plate, a support structure, bearings, and stoppers (Figure 2(a)). The base plate was a solid steel plate. Three metal tubes, a wall, and two safety stoppers were securely bolted to the base plate. The stoppers were placed to prevent the pendulum from swaying beyond the normal ankle joint range and, thus, reduced the risk of injury to the subject from excessive ankle joint movements. The moving components of the IPSA included the main shaft, end shaft, foot plates, and inverted pendulum (Figure 2(b) and 2(c)). The main shaft had two sections: a 2 in. (5.08 cm) diameter section and a 4 in. (10.16 cm) diameter section. The latter served as the support into which the inverted pendulum was inserted and locked into place in an upright position. Two bearings supported the main shaft and one bearing supported the end shaft. The foot platform, held in place by two side supports, acted as the base for the foot plates onto which the subject's feet were placed. The two foot plates were separated by 15 cm and were bolted to the foot platform. The feet were secured to the foot plates using Velcro straps.

The inverted pendulum was designed so that its mass and the location of its center of mass could be changed. The mass was changed by stacking lifting weights onto the pendulum as shown in Figure 1, and spacers could be used to translate the added mass to varying heights in 1 in. (2.54 cm) increments. Without any weights, the pendulum weighed 17.2 kg but was designed to hold a maximum of 90 kg of additional weight, that is, a total maximum weight of 107.2 kg. In order to determine the total moment of inertia of the moving components of the IPSA about the common rotational axis, the moments of inertia of the inverted pendulum, shafts, foot platform, and side supports were individually computed and then summated, resulting in a total moment of inertia of 4.98 kg·m^2^.

A laser displacement sensor (LK-2500, Keyence, Japan) with an accuracy of 10 *μ*m was utilized to measure the angle of the IPSA's inverted pendulum. A reaction torque sensor (TS11-200, Durham Instruments, Germany) with an accuracy of 0.05 Nm was implemented to record the resultant torque at the rotational axis of the inverted pendulum.

### 2.2. Experimental Performance Testing of IPSA

FES was applied by means of an electrical stimulator (Compex Motion, Compex SA, Switzerland). 5 cm × 5 cm electrodes (StimTrode, Axelgaard Co., Ltd., USA), coated with hypoallergenic gel, were used to deliver the electric current to the nerves innervating the plantarflexors. Two channels of stimulation were used, one channel for each plantarflexor (right and left legs). Note that the dorsiflexors (e.g., tibialis anterior muscles) were not required to be activated since only plantarflexors are active during natural quiet standing as the body accelerates forward in the natural standing position [20]. When larger body sway fluctuations are expected (such as during perturbed standing), the dorsiflexors should be stimulated as well.　The electrodes for the plantarflexors were placed along the midline of the posterior side of the calf muscles. The anode was placed above the gastrocnemius muscles and the cathode above the Achilles tendon. A rectangular, biphasic, asymmetric, charge-balanced, and monopolar stimulation waveform with a pulse duration of 300 *μ*s and a stimulation frequency of 40 Hz were used in the experiments. The stimulation current was either fixed or controlled by the applied PD controller, depending on the experimental conditions described below.

Three healthy and young individuals participated in this study (Subject A: age 22 yrs, height 180 cm, and weight 80 kg; Subject B: age 26 yrs, height 180 cm, and weight 83 kg; Subject C: age 21 yrs, height 172 cm, and weight 60 kg). Each subject gave written informed consent to the experimental procedure, which was approved by the local institutional ethics committee in accordance with the Declaration of Helsinki on the use of human subjects in experiments. Four conditions were tested with each subject: (1) no stimulation (i.e., voluntary control of the ankle muscles) (NOstim); (2) a PD controller with a larger derivative gain (PDstimLD); (3) a PD controller with a smaller derivative gain (PDstimSD); and (4) stimulation with a constant current (CONSTstim). “Adequate” controller gains for PDstimLD were selected based on our previous finding that the postural control system during quiet stance adopts a control strategy that relies heavily on velocity information [22, 23]. “Inadequate” controller gains for PDstimSD were determined by reducing the derivative gain arbitrarily. Each task lasted for 60 seconds or until the pendulum movement was interrupted by the safety stoppers. Three trials were performed for each condition. For the voluntary condition, the subject voluntarily stabilized the IPSA. In the two closed-loop conditions (PDstimLD and PDstimSD), a PD control strategy was employed with a constant offset value corresponding to the gravity toppling torque of the IPSA (Table 1). In CONSTstim, the average stimulation intensity during PDstimLD was used as the constant stimulation intensity (Table 1). The first trials were performed in the order of NOstim, PDstimLD, PDstimSD, and CONSTstim to identify the stimulation intensity for CONSTstim, whereas the remaining 2∗4 trials were randomized. Sufficient resting time was provided in between two trials. Prior to the testing, the subjects were allowed to familiarize themselves with both the IPSA and the electrical stimulation. Since the subjects were mechanically supported by the standing frame, the plantarflexors can be completely relaxed during standing [17]. For the FES conditions, subjects were instructed not to use voluntary efforts to control the inverted pendulum. Although we believe that voluntary and reflexive contributions to controlling the pendulum were minimal during the FES tasks, this was not verified.

The pendulum angle and torque (sampled at 100 Hz and low-pass filtered using an 8th-order Butterworth filter with a cutoff frequency of 10 Hz), measured using a customized LabVIEW program (version 8.0, National Instruments, TX, USA), were used to assess the performance within each condition. In cases of successfully stabilized trials (i.e., trials that lasted for 60 seconds), we calculated the root mean square (RMS) of the measured pendulum angle and torque and compared the averages of the three trials per subject across the different conditions.

## 3. Results

**Figure 3:**
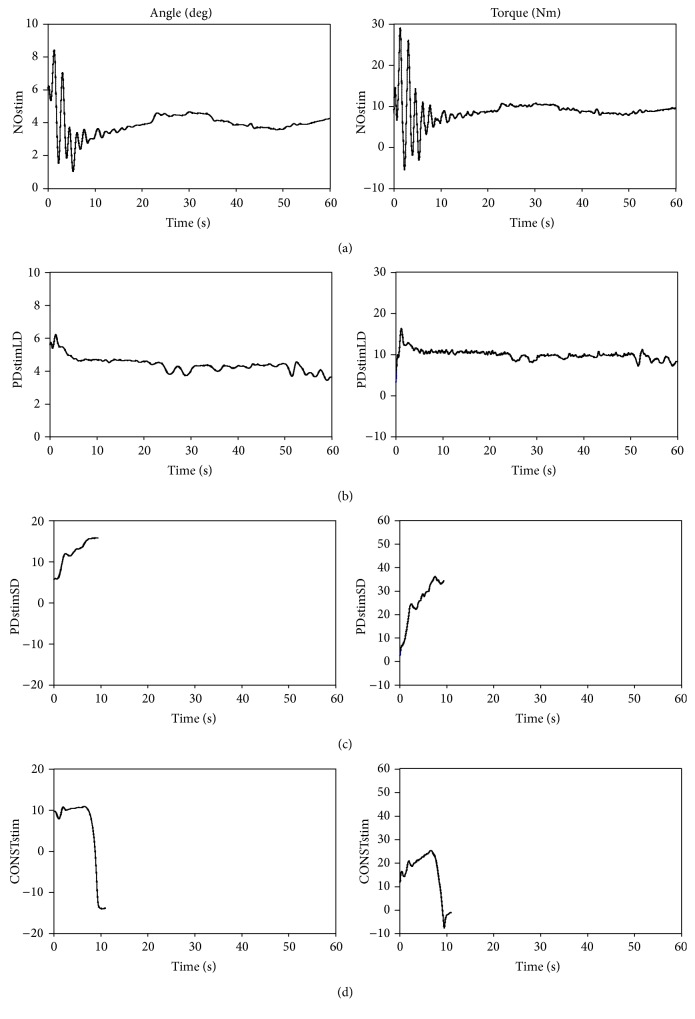
Examples of the pendulum fluctuation (angle and torque) during the four different conditions (single subject): (1) voluntary control—NOstim (top graph); (2) adequate controller—PDstimLD (second graph from top, using a P-gain of 100 Nm/rad and a D-gain of 85 Nm·s/rad); (3) inadequate controller—PDstimSD (third graph from top, using a P-gain of 100 Nm/rad and a D-gain of 30 Nm·s/rad); and (4) constant stimulation—CONSTstim (bottom graph, using a stimulation amplitude of 33 mA). The left column shows the angular displacements and the right column the measured torque. Positive angles and torques in the panels correspond to plantarflexor contractions of the subject. For the CONSTstim and PDstimSD conditions, the pendulum movement was interrupted by the built-in safety stoppers after approximately 10 seconds into the trials.

Figure 3 exemplifies the angle and torque fluctuation of the pendulum during the four different conditions. For the CONSTstim and PDstimSD conditions, the generated ankle torque was inadequate for stabilizing the system, causing the pendulum to drop. The unstable behavior was observed for all 18 trials (3 subjects, 3 trials, and 2 conditions) associated with these two conditions. The time duration from trial start to pendulum drop was 8.10 ± 2.12 seconds for CONSTstim and 13.21 ± 3.23 seconds for PDstimSD (mean ± one standard deviation for all subjects).

For both the NOstim and PDstimLD conditions, the generated ankle torque dynamically stabilized the pendulum for the entire trial length of 60 seconds. This behavior was observed for all 18 trials (3∗3∗2) associated with these two conditions.  Table 2 presents the standard deviations of the measured angle fluctuation for all subjects and both conditions. The standard deviations indicate that the pendulum in PDstimLD was stabilized in a similar manner as in NOstim. These experiments demonstrate that, by examining the angle and torque fluctuations of the IPSA pendulum, we can evaluate the ability of the controller to adequately regulate plantarflexor activity.

## 4. Discussion

The purpose of this study was twofold. First, we reported on the design and setup of IPSA. Second, we tested IPSA, showing that it ensures the safety of the subject and at the same time keeps the subject in the standing posture as required for evaluating FES control systems. We demonstrated that PDstimLD stabilized the subject's body sufficiently well (i.e., equivalently well to NOstim) while PDstimSD and CONSTstim did not, probably due to the use of inappropriate control strategies (i.e., insufficient amount of derivative gain or not facilitating time-varying calf muscle contractions). These results suggest that IPSA can be a useful platform for designing FES control systems in a well-controlled manner. That is, using the IPSA, we can directly assess the performance of a particular FES controller for ankle joint regulation while eliminating the effect of contributions from other body segments. Using the IPSA for studying different FES control strategies for standing has the following benefits: (1) supported by the standing frame, individuals with paralysis in their lower limbs can safely participate in the testing; (2) since the standing frame allows the lower limb muscles to be completely relaxed during standing [17], also able-bodied individuals can participate in the testing; (3) since the weight of the inverted pendulum can be gradually increased, the performance of different FES control strategies can be tested without the risk of damaging joints and/or falling; and (4) in contrast to testing an FES control strategy in supine or prone position, we can accurately replicate natural muscle damping and stiffness during quiet standing, which has been shown to significantly contribute to the control mechanism of that postural task [21, 24].

Also Loram's team designed an inverted pendulum that was stabilized via ankle torque modulation during standing [18, 19]. While their system was capable of measuring pendulum torque and angle as well, their body support mechanism did not include passive knee stabilization for ensuring complete elimination of natural plantarflexor activity during standing [17]. In addition, the device did not feature safety stoppers and lacked flexibility with respect to weight application. These differences are not surprising as their system was not intended to be used for developing FES control strategies in different populations but rather to study human balance control in healthy people.

In this study, we solely focused on the ankle joint and its movement in the anterior-posterior direction using an artificial inverted pendulum. The reason for choosing the ankle joint was that it plays a key role in maintaining balance of the entire body, being the closest joint to the contact surface during standing [17, 22–25]. In addition, we adopted an artificial pendulum rather than a human-body pendulum (with braces for all joints except for the ankle joint [4]) in order to ensure the rigidity of the pendulum. Such a setup also allows researchers to modify the load relatively easily, which is beneficial for investigating adequate FES control strategies. However, to realize an FES controller for arm-free standing, it is obvious that we need to also examine adequate FES control strategies for other joints (such as knee and hip [7, 8, 14]) and other degrees of freedom (such as the medial-lateral direction [25]). For this purpose, more sophisticated systems such as the multipurpose rehabilitation frame developed by Matjacić et al. [26] are more adequate as they allow studying other joints and joint activity in lateral movements. Another challenge that could be encountered when developing a practical FES controller for arm-free standing is the effect of residual muscle activity, which could happen, for example, in case of individuals with incomplete spinal cord injury. As the IPSA with its standing frame provides an environment that allows zero muscle activity [17], it can be used to develop FES controller without residual muscle activity. At the same time, residual muscle activity, if present, can pose an unexpected disturbance for the developed FES controller. While there is no conclusive solution for this issue at this time, we demonstrated that a PD control strategy facilitated a patient's stability during quiet standing who exhibited residual muscle activity [13]. Nevertheless, future studies are needed to investigate its effect in more detail.

## 5. Conclusion

The IPSA was developed to help engineers and scientists test, characterize, and select controllers that are suitable for closed-loop FES control. As demonstrated in this paper, the IPSA represents an adequate platform for studying FES control of standing in general and for ankle joint regulation in particular. However, the IPSA can also be used for examining FES control strategies intended for other applications and for the regulation of other joints, for example, to characterize temporal dynamics, noise rejection, or fatigue compensation during FES.

## Figures and Tables

**Table 1:** Gains for the PD controller in the PDstimLD and PDstimSD conditions and stimulation intensity in the CONSTstim condition. P-gain stands for the proportional gain and D-gain stands for the derivative gain of the PD controller.

Subject	PDstimLD gains		PDstimSD gains		CONSTstim
	P-gain [Nm/rad]	D-gain [Nm·s/rad]	P-gain [Nm/rad]	D-gain [Nm·s/rad]	Intensity [mA]
A	100	85	100	30	33
B	110	80	110	50	33
C	70	60	70	20	34

**Table 2:** The average standard deviation values of the pendulum angle and torque fluctuations for the PDstimLD and NOstim conditions for each subject. Each value represents the mean ± one standard deviation for three trials in each condition.

Subject	Angle standard deviation [deg]		Torque standard deviation [Nm]	
	PDstimLD	NOstim	PDstimLD	NOstim
A	0.504 ± 0.222	0.382 ± 0.121	1.38 ± 0.55	0.96 ± 0.23
B	0.565 ± 0.194	0.621 ± 0.510	1.41 ± 0.48	1.64 ± 1.21
C	1.486 ± 0.411	0.418 ± 0.126	3.55 ± 1.09	1.05 ± 0.29

## Abbreviations

CONSTstim: Stimulation condition with a constant current
FES: Functional electrical stimulation
IPSA: Inverted Pendulum Standing Apparatus
NOstim: No stimulation condition
PD: Proportional-derivative
PDstimLD: Stimulation condition with a PD controller with a large derivative gain
PDstimSD: Stimulation condition with a PD controller with a small derivative gain
RMS: Root mean square.
